# Supplementary figures and images for: Sulfamethoxazole-trimethoprim plus rifampicin combination therapy for methicillin-resistant Staphylococcus aureus infection: An in vitro study
Source: PLoS One. 2025 May 20;20(5):e0323935. doi: 10.1371/journal.pone.0323935 (PMC12091750; doi:10.1371/journal.pone.0323935)

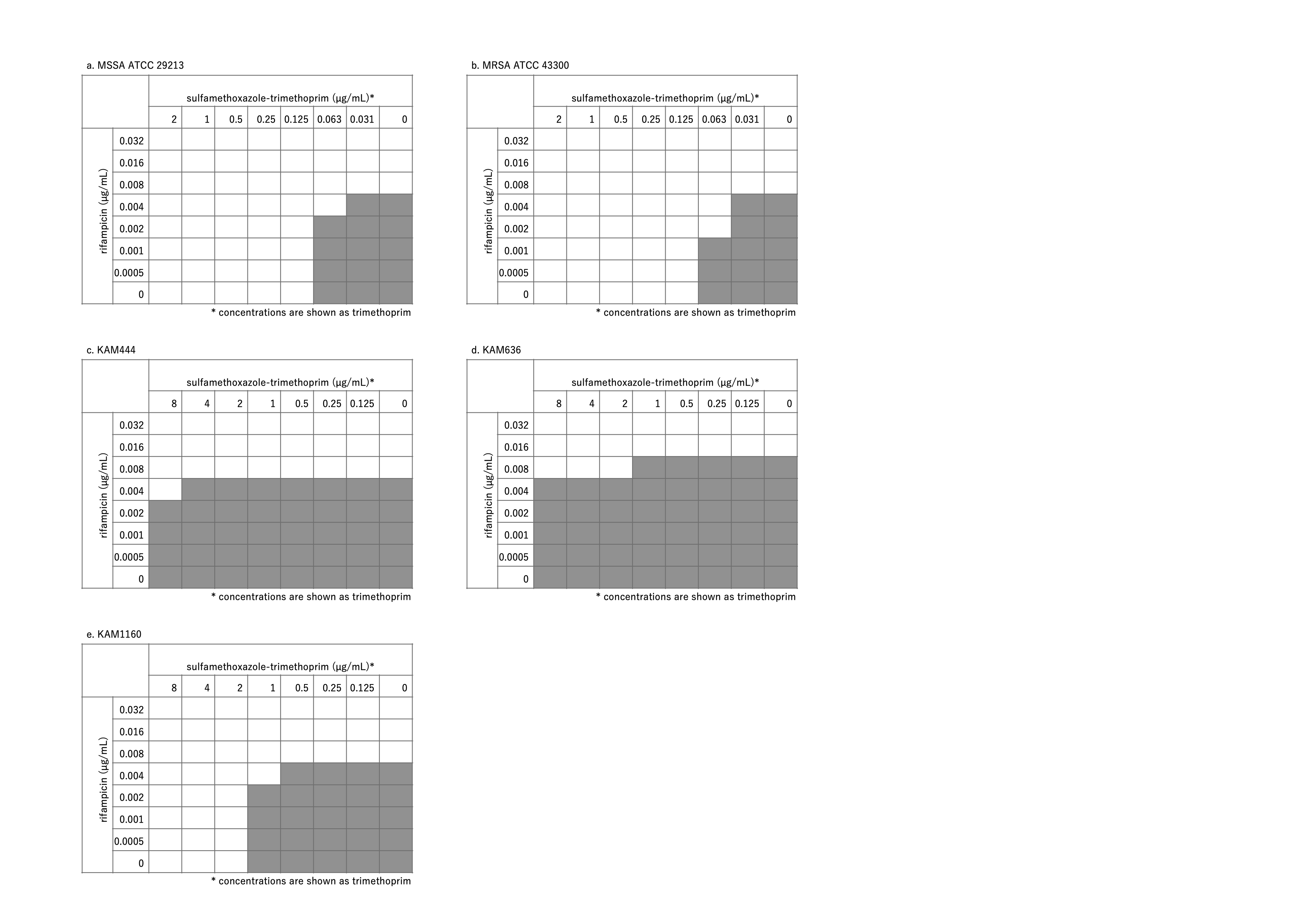

Supplement: S1 Fig — The gray wells indicate bacterial growth, and the blank wells indicate the inhibition of bacterial growth. ATCC29213 (a) and ATCC43300 (b) are reference strains of MSSA and MRSA, respectively. KAM444 (c) and KAM636 (d) are ST-R, and KAM1160 (e) is ST-LS MRSA isolates. (TIF) [file pone.0323935.s001.tif]
