# Supplementary material for: Sulfamethoxazole-trimethoprim plus rifampicin combination therapy for methicillin-resistant Staphylococcus aureus infection: An in vitro study
Source: PLoS One. 2025 May 20;20(5):e0323935. doi: 10.1371/journal.pone.0323935 (PMC12091750; doi:10.1371/journal.pone.0323935)
Supplement: S1 Table — The combination represents sulfamethoxazole-trimethoprim and rifampicin therapy, and alone represents sulfamethoxazole-trimethoprim or rifampicin therapy. (DOCX) [file pone.0323935.s002.docx]

**S1 Table. Simulator and actual concentrations (μg/mL) of sulfamethoxazole, trimethoprim and rifampicin.**

|  |  | Drugs combination or alone | Sampling times (hr) | | | | | | | | | | | |
| --- | --- | --- | --- | --- | --- | --- | --- | --- | --- | --- | --- | --- | --- | --- |
|  |  |  | 0 | | 2 | | 4 | | 8 | | 12 | | 24 | |
| Sulfamethoxazole | |  |  |  | |  | |  | |  | |  | |  |
|  | Simulated concentrations |  | 0 | | 43.23 | | 47.03 | | 35.63 | | 24.80 | | 32.97 | |
|  | Actual concentrations in KAM636 sample (%) | combination | 0 | | 40.11 (93) | | 44.83 (95) | | 33.79 (95) | | 22.77 (92) | | 31.47 (95) | |
|  |  | alone | 0 | | 40.65 (94) | | 45.56 (97) | | 35.15 (99) | | 22.24 (90) | | 32.97 (100) | |
| Trimethoprim | |  |  |  | |  | |  | |  | |  | |  |
|  | Simulated concentrations |  | 0 | | 2.28 | | 2.48 | | 1.88 | | 1.31 | | 1.74 | |
|  | Actual concentrations in KAM636 sample (%) | combination | 0 | | 2.45 (107) | | 2.66 (108) | | 2.06 (110) | | 1.48 (114) | | 1.92 (110) | |
|  |  | alone | 0 | | 2.33 (102) | | 2.69 (109) | | 2.11 (112) | | 1.46 (112) | | 1.91 (110) | |
| Rifampicin | |  |  |  | |  | |  | |  | |  | |  |
|  | Simulated concentrations |  | 0 | | 8.00 | | 5.82 | | 1.87 | | 0.54 | | 0.01 | |
|  | Actual concentrations in KAM636 sample (%) | combination | 0 | | 7.94 (99) | | 5.62 (97) | | 1.66 (89) | | 0.59 (110) | | ND^a^ | |
|  |  | alone | 0 | | 7.88 (99) | | 5.35 (92) | | 1.65 (88) | | 0.60 (112) | | ND^a^ | |

^a^ND: Not detected (<0.01 µg/mL)

The combination represents sulfamethoxazole-trimethoprim and rifampicin therapy, and alone represents sulfamethoxazole-trimethoprim or rifampicin therapy
