# Supplementary material for: Sulfamethoxazole-trimethoprim plus rifampicin combination therapy for methicillin-resistant Staphylococcus aureus infection: An in vitro study
Source: PLoS One. 2025 May 20;20(5):e0323935. doi: 10.1371/journal.pone.0323935 (PMC12091750; doi:10.1371/journal.pone.0323935)
Supplement: S2 Table — (DOCX) [file pone.0323935.s003.docx]

**S2 Table. Actual concentrations (μg/mL) of sulfamethoxazole, trimethoprim and rifampicin in each strain study.**

|  | Drugs combination or alone |  | Sampling times (hr) | | |
| --- | --- | --- | --- | --- | --- |
| Strain |  |  | 2 | 4 | 8 |
| MSSA ATCC 29213 | combination | Sulfamethoxazole | 38.40 | 42.83 | 32.67 |
|  |  | Trimethoprim | 2.21 | 2.38 | 1.76 |
|  |  | Rifampicin | 7.95 | 5.67 | 1.68 |
|  | alone | Sulfamethoxazole | 40.39 | 45.31 | 34.58 |
|  |  | Trimethoprim | 2.18 | 2.42 | 1.93 |
|  |  | Rifampicin | 8.35 | 5.61 | 1.65 |
| MRSA ATCC 43300 | combination | Sulfamethoxazole | 45.39 | 49.16 | 34.23 |
|  |  | Trimethoprim | 2.45 | 2.66 | 1.90 |
|  |  | Rifampicin | 7.72 | 5.51 | 1.64 |
|  | alone | Sulfamethoxazole | 42.15 | 47.01 | 35.60 |
|  |  | Trimethoprim | 2.22 | 2.50 | 1.95 |
|  |  | Rifampicin | 8.29 | 5.83 | 1.77 |
| KAM444 | combination | Sulfamethoxazole | 41.29 | 45.77 | 34.60 |
|  |  | Trimethoprim | 2.31 | 2.58 | 1.92 |
|  |  | Rifampicin | 7.75 | 5.46 | 1.68 |
|  | alone | Sulfamethoxazole | 42.57 | 47.36 | 36.29 |
|  |  | Trimethoprim | 2.37 | 2.63 | 1.90 |
|  |  | Rifampicin | 7.79 | 5.53 | 1.64 |
| KAM 636 | combination | Sulfamethoxazole | 40.11 | 44.83 | 33.79 |
|  |  | Trimethoprim | 2.45 | 2.66 | 2.06 |
|  |  | Rifampicin | 7.94 | 5.62 | 1.66 |
|  | alone | Sulfamethoxazole | 40.65 | 45.56 | 35.15 |
|  |  | Trimethoprim | 2.33 | 2.69 | 2.11 |
|  |  | Rifampicin | 7.88 | 5.35 | 1.65 |
