# Supplementary material for: Sulfamethoxazole-trimethoprim plus rifampicin combination therapy for methicillin-resistant Staphylococcus aureus infection: An in vitro study
Source: PLoS One. 2025 May 20;20(5):e0323935. doi: 10.1371/journal.pone.0323935 (PMC12091750; doi:10.1371/journal.pone.0323935)
Supplement: Attachment — Submitted filename: 250416_Responses_to_Reviewer.docx [file pone.0323935.s005.docx]

**Response to Reviewers**

We sincerely thank the Editor and reviewers for their thorough evaluation of our manuscript and constructive feedback. We have revised the manuscript accordingly. Below we provide a point-by-point response to each comment. The reviewer’s comments are quoted **in italics**, and our responses follow each comment.

**Response to Editor**

**Major Point 1: Sample Size and Strain Selection**

**Comment (Editor):** *“Expand your study to include a larger sample size, encompassing both clinical and reference strains. This will strengthen the validity of your findings.”*

**Response:** We agree with the importance of a broader sample. In the revised manuscript, we have added one additional **ST-low-susceptible MRSA clinical isolate (KAM1160)** to address the small sample size. We have explicitly noted in the Discussion section that the limited strain selection is a **study limitation**, and we emphasize the need to examine more strains in future studies.

**Major Point 2: Literature Review**

**Comment (Editor):** *“Re-evaluate your claim regarding the lack of in vitro studies for the TMP/SMX + rifampin combination in pharmacokinetic/pharmacodynamic models. A reviewer has pointed out at least one relevant study (El Haj et al., 2018) that you should acknowledge and discuss.”*

**Response:** Thank you for this suggestion. We have revised the manuscript to acknowledge the prior in vitro study by **El Haj et al. (2018)** that examined the trimethoprim-sulfamethoxazole (TMP/SMX) + rifampin combination in a pharmacokinetic/pharmacodynamic (PK/PD) biofilm model. In the Discussion section of the revised manuscript, we now cite this study and compare our methods and findings with those of El Haj et al.

**Major Point 3: Guidelines**

**Comment (Editor):** *“Broaden your discussion of guidelines to include international perspectives, not just those from Japan. This will increase the relevance of your work for a global audience.”*

**Response:** We have expanded our manuscript to include international guidelines as suggested. In addition to the Japanese guidelines (2019) that were originally cited, the Introduction and Discussion of the revised manuscript now reference **key international guidelines** on MRSA treatment. Specifically, we have added the **2011 Infectious Diseases Society of America (IDSA) clinical practice guidelines** for the treatment of MRSA infections, which mention oral combination therapy with ST plus RFP for certain indications (e.g., bone and joint infections). We also cite the **2021 UK MRSA treatment guidelines**, which note that while RFP combinations were once considered (especially for biofilm-related infections), there is no clear evidence supporting their efficacy in that context. Furthermore, as noted by the Editor, we have included the **European Pediatric Bone and Joint Infection Guidelines** which recommend ST plus RFP in specific scenarios. By incorporating these sources, we have ensured that our discussion is internationally relevant and provides a more comprehensive perspective beyond the Japanese context.

**Major Point 4: Strain Characterization (MIC Discrepancies)**

**Comment (Editor):** *“Clarify the discrepancies in MIC values for your clinical strains, particularly regarding the strain with an intermediate MIC to TMP/SMX. Discuss the implications of these differences on your results and conclusions.”*

**Response:** We investigated the noted discrepancies in minimum inhibitory concentration (MIC) values by performing **additional experiments** for clarification. We reconfirmed the MIC of ST for each clinical isolate using both the standard microdilution method and the E-test, alongside the quality control strain (ATCC 29213) to ensure accuracy of the methods.

For **strain KAM636**, which originally appeared to have an intermediate MIC of ST, the E-test revealed slight bacterial growth within the inhibition zone, corresponding to an MIC of **16 µg/mL**. This finding explains the previous discrepancy, as 16 µg/mL indicates a **resistant** level (above the susceptibility breakpoint). We have corrected the reported MIC of KAM636 in the revised manuscript to 16 µg/mL and clarified that this strain is ST-resistant.

For the **newly added clinical isolate KAM1160** (ST-low-susceptible strain), the E-test yielded an MIC of **3 µg/mL**. According to the E-test manufacturer’s instructions, when an MIC falls between two standard dilution values, it should be rounded up to the higher two-fold concentration. Therefore, 3 µg/mL is interpreted as 4 µg/mL in broth microdilution equivalence, which falls into the resistant category (non-susceptible). In the revised manuscript, we now describe KAM1160 as a **“ST low-susceptible (ST-LS) strain,”** reflecting its borderline resistance.

We have updated the Results and Discussion sections to explain these MIC differences and their implications. Importantly, both clinical strains tested are now clearly identified as not fully susceptible to ST (one intermediate/low-susceptible and one resistant), and we discuss how the combination therapy efficacy was observed particularly in these non-susceptible strains. These clarifications ensure that our conclusions appropriately consider the variability in MIC results.

**Major Point 5: Resistance Development**

**Comment (Editor):** *“Address whether you checked for the development of rifampin resistance in your experiments, particularly for the KAM444 strain that regained growth.”*

**Response:** We have addressed this important point by analyzing whether rifampin resistance emerged during our chemostat model experiments. For **each clinical isolate**, we measured the MICs of ST and RFP for bacteria surviving after the 24-hour chemostat experiment. We found that in all cases the **MIC of RFP increased substantially after exposure to rifampin alone**, indicating that RFP resistance was indeed induced during the experiment (RFP monotherapy led to resistant subpopulations). We have added these findings to the Results and have expanded the Discussion to note that the development of RFP resistance is a significant concern when RFP is used alone, which further supports the rationale for combination therapy in our study.

**Major Point 6: Clinical Relevance**

**Comment (Editor):** *“Provide a more nuanced discussion of the clinical applicability of your findings, particularly regarding the use of this combination therapy for TMP/SMX-resistant strains and in cases of sepsis or bacteremia.”*

**Response:** We appreciate this suggestion and have revised the Discussion to more carefully frame the clinical relevance of our findings. **First**, we emphasize that our study is based solely on in vitro experiments, and thus we do **not** intend to directly recommend changes to current clinical treatment practices at this stage.

**Second**, we have added a nuanced perspective on potential clinical applicability. Specifically, we discuss that the **ST plus RFP combination** might be considered as a **future therapeutic option in certain limited scenarios** – for example, in situations where an MRSA infection is suspected but the organism’s ST susceptibility is unknown (and obtaining rapid MIC results is not feasible), and an effective oral regimen is needed (such as for step-down therapy or when IV treatment is not possible). In summary, the revised discussion highlights the potential value of our findings as a basis for future research; however, further data accumulation will be necessary before these findings can be applied clinically.

**Minor Point 1: Figure Quality**

**Comment (Editor):** *“Improve the quality of Figure 1, which was reported as blurry.”*

**Response:** We apologize for the suboptimal quality of Figure 1 in the initial submission. To resolve this issue, we have replaced Figure 1 with a **high-resolution version** in the revised manuscript.

**Minor Point 2: Language and Formatting**

**Comment (Editor):** *“Address the specific language and formatting suggestions made by the reviewers, including: (a) Removing the word ‘therapy’ where inappropriate, (b) Correcting alignment issues in Table 2, (c) Reconsidering certain assertive statements, (d) Using passive forms where appropriate, (e) Properly referencing laboratory products.”*

**Response:** We have made all the recommended language and formatting corrections in the revised manuscript:

- **a. Use of “therapy”:** We removed the word “therapy” in contexts where it was inappropriate for an in vitro study (specifically, we deleted “therapy” on original manuscript lines 44 and 312, where its use implied clinical treatment).
- **b. Table 2 alignment:** We corrected the alignment problems in **Table 2**. All columns and entries in Table 2 are now properly aligned and formatted consistently.
- **c. Assertive statements:** We revised an overly assertive sentence in the Discussion (original lines 273–274). The wording has been toned down to a more cautious statement, reflecting the fact that our conclusions are based on in vitro results with a limited number of strains.
- **d. Passive voice:** We have adjusted the manuscript text to use passive voice where appropriate to improve the formal tone.
- **e. Referencing laboratory products:** We have added proper references or source information for all laboratory products and reagents mentioned in the Methods.

We believe these changes have improved the clarity and readability of the manuscript in line with the reviewers’ suggestions.

**Additional Suggestion 1: Number of Replicates**

**Comment (Editor):** *“Explain why only two replicates were conducted instead of three.”*

**Response:** We appreciate the reviewer’s comment regarding experimental replicates. All experiments initially demonstrated high reproducibility with two replicates, supporting the reliability of our data. To enhance robustness, **the revised manuscript includes a third replicate for the MIC assay and checkerboard assay**, which confirmed consistency. For the chemostat model, due to the demanding nature of the 24-hour protocol, two replicates were conducted, both yielding highly similar results. These details are clearly described in the revised manuscript.

**Additional Suggestion 2: Removal of a Sentence**

**Comment (Editor):** *“Consider removing or revising the sentence about a previous report (lines 260-261) as suggested by Reviewer 1.”*

**Response:** We have removed the sentence in the Discussion referring to a “previous report” (along with the associated reference 19) as suggested.

**Additional Suggestion 3: Referencing Web Address**

**Comment (Editor):** *“Add the web address mentioned on line 267 to your reference list.”*

**Response:** In accordance with this suggestion (and Reviewer 1’s comment), we have added the previously unreferenced **web address (from line 267)** as a formal citation in the reference list (Ref. 23).

**Response to Reviewer #1**

**Reviewer #1:** *“The study is generally well-planned and the manuscript is generally well-written. I have few recommendations about the manuscript.”*

**Response:** We thank Reviewer #1 for the positive feedback on our study design and writing. We appreciate the specific recommendations and have addressed each point in the revised manuscript, as detailed below:

**Comment 1 (Lines 44 and 312):** *“Remove the word ‘therapy’ because this is not a clinical study.”*
**Response:** We have removed the word **“therapy”** from the relevant sentences in the manuscript (original lines 44 and 312), as suggested. We agree that since our work is an in vitro study and not a clinical trial, using the term “therapy” in those contexts was inappropriate.

**Comment 2 (Line 47):** *“Write ‘against’ instead of ‘therapy of’.”*
**Response:** We have revised the phrasing at line 47 in the manuscript. The phrase “the therapy of” has been replaced with **“against”** so that the sentence now correctly reads with “against” (e.g., “activity against MRSA”) rather than implying “therapy.”

**Comment 3 (Table 2):** *“Some alignment problems exist in some columns of Table 2. These should be corrected.”*
**Response:** We have corrected the alignment issues in **Table 2** of the manuscript. All columns in Table 2 are now properly aligned.

**Comment 4 (Lines 260-261):** *“I recommend the authors remove the sentence ‘In a previous report, RFP…’ and reference 19. In the cited report, although rifampicin and minocycline considerably decreased both WT and SCVs, both bacterial counts recovered to the initial number 48 hours later.”*
**Response:** We agree with this recommendation. We have **removed the sentence in question (about the previous report on rifampicin and minocycline)** as well as the corresponding reference 19 from the Discussion. As the reviewer noted, that cited study’s findings (rifampicin + minocycline reducing MRSA counts only temporarily) did not directly support our discussion, and omitting it has made our narrative more concise and clear.

**Comment 5 (Line 267):** *“A number can be given for the web address and this reference can be added to the references list.”*
**Response:** We have added a numbered citation for the **web address mentioned in line 267** of the original manuscript. Specifically, the web resource is now included as an entry in the reference list, and we cite it in the text by its reference number.

**Comment 6 (Lines 273-274):** *“This sentence is a bit assertive. This was an in vitro study and the number of strains tested in this study was low. You can use a more appropriate sentence or you should support this sentence with information obtained from clinical studies.”*
**Response:** We appreciate this comment and have revised the sentence in lines 273–274 to **tone down the assertiveness**. In the original manuscript, that sentence overstated the implications of our findings. We have rewritten it to be more cautious and context-appropriate, given that our evidence comes from an in vitro study with a small sample of strains.

**Response to Reviewer #2**

**Reviewer #2:** *[General comment]* “The article presents some intriguing findings, yet it is imperative to substantiate these outcomes with a more extensive sample size, encompassing both clinical and reference strains.”

**Response:** We thank Reviewer #2 for the overall positive view of our findings and the important suggestions. We have implemented changes to address the concerns raised. Below are our point-by-point responses to each comment:

**Comment 1: Sample size of strains**
*“The article presents some intriguing findings, yet it is imperative to substantiate these outcomes with a more extensive sample size, encompassing both clinical and reference strains.”*
**Response:** We completely agree that a larger sample size would strengthen the study. In response, we have added an additional **MRSA clinical isolate** to our experiments. Specifically, we included one more ST-low-susceptible MRSA clinical strain (KAM1160) and repeated the key experiments for combination efficacy with this strain. We have incorporated these new results into the revised manuscript. We have also explicitly acknowledged in the Discussion that the total number of strains tested is still small, and we have listed the limited sample size as a **limitation of our study**.

**Comment 2:** *“In the event of a resubmission, other factors will have to be taken into consideration.”*
**Response:** We understand the reviewer’s remark and agree that there are additional factors which could be important to explore in subsequent studies (for example, different strain backgrounds, resistance mechanisms, or in vivo conditions). For the current revision, we focused on the specific major points raised by all reviewers.

**Comment 3:** *“It is preferable to utilize passive forms.”*
**Response:** We have reviewed the manuscript for tone and voice, and we have revised many sentences to use the **passive voice** where appropriate.

**Comment 4:** *“Please clarify why only two replicates were conducted, rather than three.”*
**Response:** We thank the reviewer for this important comment. We have clarified this point in the revised Methods and Results. Most experiments initially showed high reproducibility with two replicates. To ensure robustness, **a third replicate was added for the MIC and checkerboard assays**, confirming consistent results. For the chemostat experiments, only two replicates were conducted due to the complexity and labor-intensive nature of the 24-hour protocol. Both runs showed nearly identical outcomes, supporting the reliability of the data.

**Comment 5:** *“The sources of the laboratory products in question are not adequately referenced.”*
**Response:** We have updated the manuscript to include proper source information for all laboratory products and reagents used.

**Response to Reviewer #3**

**Reviewer #3:** *“I carefully read the interesting manuscript describing the in vitro activity of TMP/SMX and rifampin combination for MRSA in 4 bacterial strains. There are limited therapeutic options for MRSA and sometimes even susceptible strains may persist antibiotic therapy. Therefore, combination therapy is often used. The methods are sound and overall, the manuscript is well written. There are, however, some issues to address.”*

**Response:** We thank Reviewer #3 for the careful reading of our manuscript and the encouraging remarks about our study’s significance and methodology. We appreciate the insightful points raised. Below we address each of the issues in detail:

**Comment 1: Prior in vitro studies of TMP/SMX + rifampin**
*“The authors claim there are no in vitro studies for the TMP/SMX + rifampin combination in pharmacokinetic/pharmacodynamic models. That is not accurate. El Haj et al. showed enhanced anti-biofilm capacity but raised concerns that rifampin resistance emerges. Combination of linezolid and rifampin protected against resistance (Cristina El Haj et al., 2018, Int J Antimicrob Agents 51:854–861).”*

**Response:** You are correct that a relevant in vitro study exists. We apologize for the oversight. In the revised manuscript, we have acknowledged the study by **El Haj et al. (2018)**, which evaluated ST plus RFP in an in vitro **biofilm** PK/PD model. We have added a citation of this work in our Discussion and discussed its findings relative to ours. In any case, as we have now stated in the manuscript, such in vitro studies on ST plus RFP are **rare** – El Haj et al. is one of the few – and our study provides complementary evidence using different in vitro approaches. We believe this addition strengthens the background and properly credits prior literature, while also highlighting the scientific value of our study in an area with limited existing data.

**Comment 2: Guidelines – include international ones**
*“The authors refer to guidelines from Japan. It would be helpful for the broader readership to refer to other local and international guidelines. These are a few examples: [Reviewer provides references: IDSA 2011 MRSA treatment guidelines; UK 2021 MRSA treatment guidelines].”*

**Response:** Thank you for this excellent suggestion. We have expanded our discussion of treatment guidelines to include **international perspectives**. In the revised Introduction and Discussion, we now reference the **2011 Clinical Practice Guidelines by the IDSA for MRSA treatment**, which cover adult and pediatric cases and mention oral therapy options including ST plus RFP for certain infections. We also cite the **updated 2021 UK guidelines for MRSA treatment** published in JAC-Antimicrobial Resistance, which provide a contrasting viewpoint: they discuss combination therapy with rifampicin but ultimately state that clear evidence for added benefit is lacking. These additions, along with the Japanese guideline we initially cited, give a more global context to our study.

**Comment 3: MIC discrepancies between the two clinical strains**
*“The authors investigated two reference ATCC strains (susceptible to both TMP/SMX and rifampin) and two clinical strains (resistant to TMP/SMX and susceptible to rifampin). These two clinical strains, however, differed in their MICs to TMP/SMX, which were 3 and >32. The EUCAST and CLSI breakpoints for TMP/SMX are >4 µg/mL for resistance and ≤2 µg/mL for susceptible. Thus, one of the clinical strains tested was actually non-susceptible with an intermediate MIC and the other was resistant. Using the checkerboard method, this intermediate strain was resistant with MIC >8 (in combination) and reduced to 2 when rifampin was added. Can the authors comment on these differences?”*

**Response:** We appreciate the reviewer’s insightful comment. KAM636 was initially considered to have an intermediate MIC to ST (3 µg/mL); however, **additional testing revealed faint growth within the inhibition zone in the E-test, indicating an MIC of 16 µg/mL**. This clarified that KAM636 is in fact a resistant strain, resolving the previously noted MIC discrepancy.

Additionally, we included a new strain, **KAM1160, as a low-susceptibility isolate** (ST MIC 3 µg/mL by E-test). According to the standard E-test protocol, MIC values that fall between two-fold dilutions are reported by rounding up; therefore, this strain is reported as having an MIC of 4 µg/mL. In the revised manuscript, we describe this strain as ST-low-susceptible MRSA.

**Comment 4: Regrowth of fully resistant strain and rifampin resistance check**
*“The lowest fractional inhibitory concentration index for combination therapy was 0.5–1, indicating an additive effect, and chemostat-based kill curves showed increased efficacy of combination therapy in the TMP/SMX-resistant strains. This was not evident for the susceptible reference strains. Still, the fully resistant strain (KAM444) regained growth (but at a slower rate) under rifampin monotherapy. Did the authors check for development of resistance to rifampin in this experiment?”*

**Response:** Thank you for this important point. **We confirmed the development of RFP resistance in our chemostat experiments** and have added these results to the revised manuscript. For all clinical strains we measured RFP MICs after the 24-hour exposure and observed marked increases, indicating resistance emergence. This explains the regrowth seen under RFP monotherapy. In contrast, no regrowth was observed under ST plus RFP combination, likely because the ST component suppressed RFP-resistant subpopulations. We have clarified this in both the Results and Discussion sections, referencing prior studies such as El Haj et al., 2018.

**Comment 5: Clinical rationale for using TMP/SMX + rifampin**
“As the authors note, TMP/SMX-resistant strains are rare and TMP/SMX alone is often used as empiric therapy when MRSA is suspected. As a clinician, I wouldn’t know why I should use TMP/SMX in combination when the isolate is actually resistant to TMP/SMX. I would definitely not think of using this combination for sepsis or bacteremia as the authors suggest.”

**Response:** We appreciate the reviewer’s clinical perspective and have clarified our position in the revised manuscript. Our in vitro findings showed that ST plus RFP may suppress ST-resistant strains in combination, but we present this as preliminary data for future research—not as a clinical recommendation.

Regarding severe infections like sepsis or bacteremia, we fully agree that standard intravenous therapies should be used. We have revised the Discussion to state explicitly that ST plus RFP is not appropriate for life-threatening MRSA infections. Instead, we suggest the combination could be considered as an oral option in limited scenarios, such as when susceptibility is unknown or an effective oral regimen is needed.

**Minor Points (Reviewer #3)**

**Minor Point 1: Figure 1 quality**
*“Figure 1 is blurry.”*

**Response:** We apologize for the unclear quality of Figure 1 in the initial submission. We have addressed this by replacing **Figure 1** with a clearer, high-resolution image in the revised manuscript. The new figure is sharp and legible. We have ensured that all figure images meet the resolution requirements to prevent any blurriness. Thank you for drawing our attention to this issue.

Once again, we thank the Editor and reviewers for their valuable feedback. We believe that the revisions have significantly improved the manuscript. We hope that our responses satisfactorily address all concerns raised. Please let us know if any further clarification is needed.
